# Perioperative oral nutritional support for patients diagnosed with primary colon adenocarcinoma undergoing radical surgical procedures -Peri-Nutri Trial: study protocol for a randomized controlled trial

**DOI:** 10.1186/s40795-022-00591-y

**Published:** 2022-09-02

**Authors:** Raila Aro, Pasi Ohtonen, Tero Rautio, Juha Saarnio, Elisa Mäkäräinen, Reetta Häivälä, Markus J. Mäkinen, Anne Tuomisto, Ursula Schwab, Sanna Meriläinen

**Affiliations:** 1 Department of Gastrointestinal Surgery, Medical Research Center Oulu, Oulu University Hospital, PL 10, 90029 OYS Oulu, Finland; 2 Division of Operative Care, Medical Research Center Oulu, Oulu University Hospital, PL 10, 90029 OYS Oulu, Finland; 3 Department of Pathology, Cancer and Translational Medicine Research Unit, University of Oulu, Oulu University Hospital and Medical Research Center Oulu, Oulu, Finland; 4 Department of Pathology, University of Oulu, Oulu University Hospital and Medical Research Center Oulu, Oulu, Finland; 5 School of Medicine, Institute of Public Health and Clinical Nutrition, Finland and Department of Medicine, Endocrinology and Clinical Nutrition, University of Eastern, Kuopio University Hospital, Kuopio, Finland

**Keywords:** Malnutrition, NRS-2002, Perioperative oral nutritional support, Colon cancer, Randomized controlled trial

## Abstract

**Background:**

Colon cancer is one of the most common cancers in Finland and worldwide. Cancer-related malnutrition is a well-known risk factor for increased morbidity and mortality after surgery, and it is associated with complications and longer hospitalizations. There are no established recommendations on how to improve the nutritional status of colon cancer patients´ during the perioperative phase. Administration of simple oral nutritional supplements has been suggested to reduce complication rates, but evidence to support this practice is scarce.

**Methods:**

The Peri-Nutri trial is a prospective, multicenter, randomized, controlled trial. Its primary endpoint is to evaluate whether perioperative oral nutritional support (ONS) decreases the number of complications during the 30-day follow-up after surgery. Secondary endpoints are to study the effect of ONS on quality of life after surgery, length of stay in institutional care, 90-day mortality rate, five-year disease-free survival and overall survival. The patients with a Nutritional risk screening 2002 (NRS-2002) questionnaire result between 2 and 5 (≥ 3 are classified at risk of malnutrition) will be randomized (1:1 ratio) into either the intervention or control group. The intervention group will receive preoperative ONS two weeks before the operation, and nutritional support will continue 10 days after the operation. The control group will not receive ONS. A total of 318 patients will be randomized into two groups and patients are followed five years.

**Discussion:**

Peri-Nutri study evaluate the impact of ONS to short-term and long-term postoperative morbidity and mortality rates of colon cancer patients undergoing curative surgery. If ONS will decrease patients´ morbidity and mortality, that has a huge impact on patients´ quality of life and also to financial cost.

**Trial registration:**

ClinicalTrials.gov, NCT03863236, Registered 25 February 2019.

**Supplementary Information:**

The online version contains supplementary material available at 10.1186/s40795-022-00591-y.

## Background

Colon cancer is one of the most common cancers both in Finland and worldwide. There were a total of 2331 new colon cancer patients in Finland in 2019. The incidence of colon cancer is 42.22/100,000 [1]. The curative treatment of colon carcinoma includes a surgical resection of the tumor and postoperative adjuvant chemotherapy when indicated. The majority of operations are completed by using laparoscopic techniques.

The prevalence of malnutrition varies between 15 and 85% of patients with cancer [2–4]. It is a well-known risk factor for increased morbidity and mortality in surgery, and it is associated with longer hospital stays and a diminished quality of life [3, 5–7]. Preoperative malnutrition in gastrointestinal cancer patients is shown to be an independent risk factor for complications after surgery [3, 6–9]. It has been shown that 39–60% of newly diagnosed colon cancer patients are at risk for malnutrition and that 4% of patients are malnourished [8, 10]. These patients were more vulnerable to complications than patients without risk (62% vs 39.8%, *p* = 0.004), and they also had a higher mortality rate (8% vs 1.6%, *p* = 0.033) [8].

Although malnutrition is common among gastrointestinal cancer patients, there are only a few randomized controlled [10–15] and prospective cohort [16–18] studies concerning perioperative nutrition and treatment outcomes. Preoperative oral nutritional support (ONS) has been suggested to decrease complication rates [13, 14, 16–19], but some studies did not find this treatment beneficial [10–12, 15].

The results of previous randomized controlled studies have varied. In a study by Kabata et al., the patients had various cancers. They reported favorable effects with preoperative ONS for gastrointestinal cancer patients with no signs of malnutrition. A 14-day-long preoperative period with an oral nutritional supplement significantly decreased severe complications [14]. Whey protein was used as an ONS prior to colorectal cancer surgery with promising results in terms of improvements in a six-minute walk test in patients prior to surgery. However, due to the small sample size, the results were not statistically significant and ONS did not improve postoperative outcomes [10]. Concordantly, two studies found no significant differences in major complications or well-being in patients after major gastrointestinal surgery [13] or after colorectal surgery [11]. In two studies, groups consisted of surgical patients with both benign and malignant diseases [12, 13]; in one study, they consisted of hepatocellular cancer patients [15]. In two studies, the nutritional state was not considered in randomization, causing possible bias to the study groups [11, 12].

There have been a few cohort studies on perioperative nutrition. In a prospective 1,085 patient cohort study with benign and malignant abdominal surgical patients, malnourished patients (NRS score at least 5) receiving either parenteral or enteral nutrition prior to gastrointestinal surgery had a lower rate of infectious complications than the controls [16]. In another prospective cohort study, hospitalized patients, including abdominal surgical patients at risk of malnutrition, benefitted from enteral nutritional support because they had fewer complications than controls, whereas the patients with a normal nutritional status did not show improvement with nutritional support [17]. In a study by Wu et al., perioperative nutritional support was revealed to be crucial in moderately and severely malnourished gastrointestinal cancer patients. Most patients (68%) received parenteral nutrition [18]. In a retrospective study of ‘non-risk’ colorectal cancer patients, who did not receive postoperative nutritional support had a higher rate of postoperative complications and a longer postoperative hospital stay [19].

A review article previously evaluated nutrition-only prehabilitation and multimodal prehabilitation (ONS with exercise) on outcomes of colorectal cancer patients undergoing surgery. They found that any prehabilitation shortened the length of hospital stay after surgery and may be a protective factor for postoperative complications [20]. A randomized controlled trial revealed that a trimodal program four weeks before surgery in colorectal cancer patients significantly increased patients’ walking capacity [10]. Nutritional counseling has also been shown to be effective in colorectal cancer patients’ nutritional intake, quality of life (QoL) and status [21, 22]. In our study, all patients are given written information about recommended nutrition for colon cancer patients before and after surgery.

There are no randomized controlled studies addressing how to improve the nutritional status of colon cancer patients with a low to moderate risk of malnutrition in the perioperative phase and whether it is possible to reduce the number of complications by administering a simple oral supplement during the perioperative period. Previous randomized controlled studies have included both colon and rectal cancer patients [10, 11, 19, 21, 22]. Rectal cancer treatment differs from colon cancer treatment. It may include neoadjuvant radiotherapy or chemoradiotherapy, which may confound the results. The sample sizes of previous studies were small and nutritional status was not taken into account during the randomization, so the study groups may have differed at baseline.

If perioperative ONS decreases morbidity and mortality after colon carcinoma surgery, it could have a significant impact on decreasing human suffering. This would also decrease financial costs by decreasing complications and hospital stays.

## Objectives

### Primary objective

The primary aim of this study is to evaluate whether perioperative ONS used two weeks prior to surgery and 10 days after surgery can decrease 30-day period postsurgical complication rates.

### Secondary objectives

Secondary aims are to investigate whether ONS improves the nutritional status of colon cancer patients, reduces 30- and 90-day mortality, shortens the stay in institutional care and improves patients’ quality of life as well as their five-year disease-free and overall survival rates and improves tolerance to adjuvant chemotherapy with fewer side effects.

## Methods

### Study setting

This study is accomplished at the Oulu University Hospital, the Kuopio University Hospital, the Seinäjoki Central Hospital and the Rovaniemi Central Hospital in Finland.

### Trial design

**Fig. 1:**
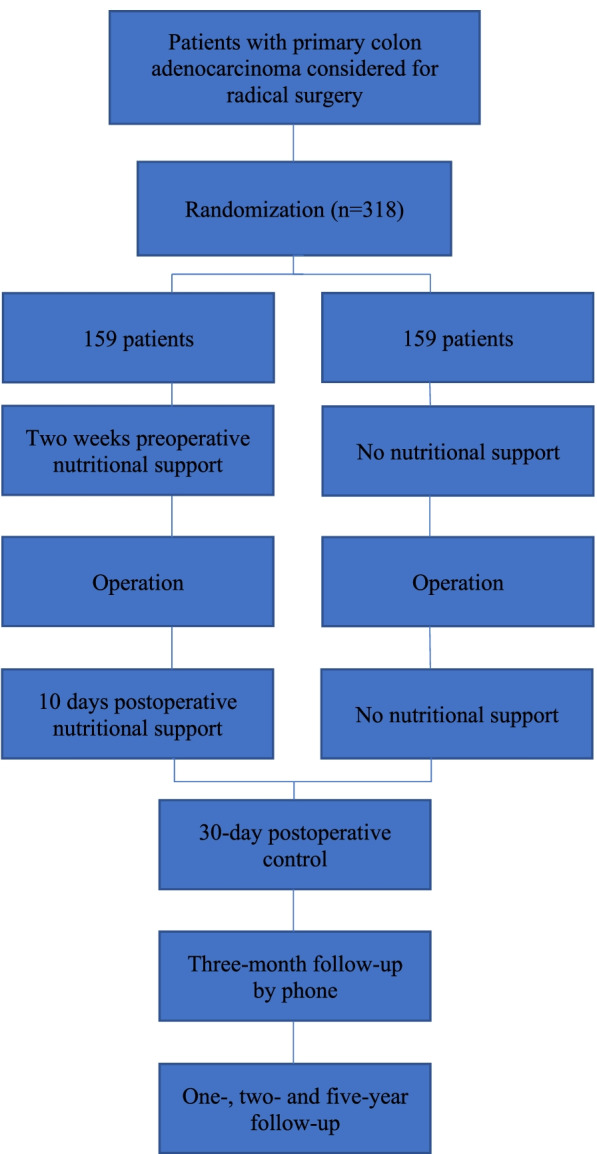
Flowchart

The Peri-Nutri trial is a prospective, multicenter, nonblinded, randomized study. Consecutive patients diagnosed with primary colon adenocarcinoma and considered for radical surgical procedures are enrolled in this study. The trial was approved by the Ethics Committee at Oulu University Hospital and registered at Clinical Trials NCT03863236. Patient recruitment started in April 2019 at the Oulu University Hospital, November 2019 at the Seinäjoki Central Hospital and in April 2021 at the Kuopio University Hospital. Recruitment will be completed on approximately April 1, 2023. The study flow is illustrated in Fig. 1. The design of this study is open label. Blinding is not possible because there is no adequate ONS-placebo product to use. Blinding is not possible for surgeons because they recruit the patients, participate in patient care and collect the data.

### Eligibility criteria

Consecutive patients examined for colon cancer at each research site will be evaluated for their suitability for inclusion in the trial. After all inclusion and exclusion criteria are verified, the patient will be informed about the study at the outpatient clinic and informed consent will be obtained.

#### Inclusion criteria

- 18 years or older
- Primary colon adenocarcinoma
- Curative operation is possible
- Patient has a life expectancy of at least 12 months
- Patient signs the informed consent and agrees to attend all study visits

#### Exclusion criteria

- Recurrent colon adenocarcinoma
- Metastatic disease
- Cancer that will require multiorgan resection
- Pregnant or suspected pregnancy
- Patients with a comorbid illness or condition that would preclude the use of surgery (ASA 5)
- Patients with concurrent or previous malignant tumors within five years before study enrollment
- Patient undergoing emergency procedures
- Dialysis
- Liver dysfunction; Child–Pugh B or worse
- NRS score < 2 or > 5
- Body mass index (BMI) under 18.5
- Weight loss of 15% or more in the past six months
- Serum albumin less than 30 without liver or renal dysfunction
- Chronic malnutrition: short bowel syndrome, previous gastrectomy, pancreatic dysfunction
- Language barrier, impaired cognition or other reasons why informed consent is not possible
- Allergy to ONS contents
- Patient not willing to participate

### Outcomes

#### Primary outcome

The primary outcome is the number of complications during the 30-day period postoperatively.

#### Secondary outcomes

Secondary outcomes are quality of life after surgery, length of stay in institutional care, 90-day mortality, tolerance to adjuvant chemotherapy and five-year disease-free and overall survival.

### Sample size

The 30-day complication rates in previous studies [10, 12–17, 23] varied from 8.2 to 77.3% (8.2 to 46.9% for patients receiving ONS, and 12.0 to 77.3% for patients without ONS). Since the sample size of those studies varied from 43 to 432 participants, we calculated weighted 30-day complication rates by using the size of the study as a weight. Accordingly, the weighted 30-day complication rate is 22.0% for patients receiving ONS and 14.9%-unit higher (i.e., 36.9%) for patients without ONS. Further, assuming a two-sided 5% significance level (alfa) and a power of 80% (1-beta) we would need 143 patients per group to show ONS being superior. Accounting for a 10% drop-out rate, the final sample size needed is 159 per group. To recruit this number of patients a 48-month inclusion period was anticipated.

### Randomization and allocation

Patients undergoing surgery for primary adenocarcinoma of the colon and fulfilling the inclusion criteria will be randomized on their preoperative visit to the outpatient clinic approximately two to three weeks prior to surgery after signing an informed consent form. The patients will be randomly allocated (1:1 ratio) to either an intervention group or a control group according to a computer-generated list compiled by a biostatistician otherwise uninvolved in the clinical care of the trial patients. Allocation will be stratified according to NRS-2002 results (2, 3–4 and 5) and blocked within strata using random permuted blocks (block sizes 2, 4, 6 and 8). A separate randomization list will be created for each participating center. All patients undergoing surgery for colon cancer will be approached and assessed for eligibility and, once their consent is obtained, randomization will be performed. The random allocation will be performed in Case Report Forms (eCRF) where the treatment group cannot be seen before both patient consent and all eligibility criteria fulfilled.

### Intervention

The control group will not receive ONS. The intervention group will receive preoperative nutritional support (Resource 2.5 Compact® or Nutridrink Compact®) two doses per day two weeks before the operation, and nutritional support will continue for 10 days after the operation.

Resource 2.5 Compact® was chosen as the ONS to ensure patient compliance because it is a protein- and energy-rich product with a small portion size. It does not contain fiber, which might cause abdominal discomfort. When Resource 2.5 Compact was no longer available, Nutridrink Compact was chosen as the ONS. The products are equal in size and contain the same amount of calories, proteins, carbohydrates and fat. All patients will be given written information about recommended nutrition for colon cancer patients going to and recovering from surgery. The nutritional recommendations are written by a local licensed nutritional therapist based on the literature and her clinical experience [24]. The information includes recommendations to use nutritionally rich, healthy foods in ways that they are easily digested and would not cause bloating or provoke bowel occlusions. Patients will be encouraged to eat vegetables, fruits, whole grains, milk products, fish and meat or plant-based proteins. Regular mealtimes will be emphasized, and it will also be highlighted not to seek to lose weight during cancer treatment. The demand for protein may be elevated (1.5 g/kg/day) during the treatment phase, and examples of how to obtain enough protein from the diet will be given to the patients.

Thus, the randomization groups will be:

1. no oral nutritional support (control group) *n* = 159
2. pre- and postoperative oral nutritional support (intervention group) *n* = 159

### Nutritional status assessment

In this study we will use several methods to analyze patients’ nutritional status. The NRS-2002 is a screening tool designed to assess the risk of malnutrition, as shown in Table 1 [25]. It takes into account BMI, weight loss during the past three months, food consumption during the past week, the severity of the illness and age under/over 70. Maximum points are 7. Patients who scored 0 are not at a nutritional risk, patients who scored 1–2 are at a low nutritional risk, and patients who scored 3–4 are at a moderate risk of malnutrition. A score of 5 or more indicates a severe risk of malnutrition. In our study, each patient will be scored a minimum 2 points at baseline and at the one-month follow-up visit because of their disease severities since these patients have local cancer and an upcoming or recent major abdominal operation.

**Table 1:** NRS-2002 screening tool

1. Nutritional situation			Points
	BMI	< 18.5	3
		18.5–20.5	2
		> 20.5	0
	Weight loss during last 3 months	weight loss < 5%	0
		weight loss 5–10%	1
		weight loss 10.1–15% (or > 5%/2 months)	2
		weight loss > 15% (or > 5%/1 month)	3
	Food amount last week	ate normal amount	0
		ate over 50% of normal	1
		ate approximately 50% or less of normal	2
		ate very sparingly	3
			max points 3
2. Severity of disease nutritionally		normal situation (no significant disorders)	0
		Mild • patient on his/her feet despite of deteriorated overall condition • chronically sick patient staying in hospital due to comorbidities • chronic wound below 25 cm^2^ • local cancer • alcohol or drug problem	1
		moderate • bedridden patient • ambulatory patient with, e.g., widespread cancer, severe inflammatory bowel disease, recent major abdominal operation, repeated operations, recent stroke, severe infection, burn injury, pressure ulcer, widespread chronic wound, hip fracture, multiple traumas, acute leukemia	2
		severe • intensive care • head injuries • stem cell transplantation • anorexia nervosa	3
3. Influence of age	Age	< 70	0
		≥ 70	1

*Abbreviations*: *BMI* Body Mass Index

Bioimpedance analysis has been shown to be reliable in analyzing the nutritional status and body composition of a patient with a cancer [26, 27]. It measures body tissue resistance and reactance, giving an estimate of total body water. When the patient’s height is known, the estimation of fat-free mass can be calculated [28, 29].

A reliable estimation of physical strength can be measured by the handgrip strength. It has also been proven to be a method for screening malnutrition, and it was also shown that patients who were at risk of malnutrition according to NRS-2002 had a lower handgrip strength [30, 31]. A low handgrip strength has been associated with increased mortality [32].

Laboratory markers have been considered an easy method for assessing malnutrition. Traditionally, the laboratory parameters albumin, CRP and total lymphocyte count have been used [33].

Computed tomography (CT) can be used to assess body composition and lean body mass. Lean body mass is described as the sum of fat-free mass. The cross-sectional area of muscle and fat at the level of L3 has been shown to correlate with the volume of tissues in the whole body [34]. Sarcopenia and myosteatosis can be calculated with a CT scan. Sarcopenia is defined as a skeletal muscle index (SMI) < 41cm^2^/m^2^ for women, 43cm^2^/m^2^ for men with BMI < 25 kg/m^2^ and 53 cm^2^/m^2^ for men with BMI ≥ 25 kg/m^2^ [35]. Myosteatosis is defined as a Hounsfield unit (HU) < 41 for patients with BMI < 25 kg/m^2^ and HU < 33 for patients with BMI ≥ 25 kg/m^2^ [35].

### Surgery

Standard treatment of colon adenocarcinoma includes either a left or right hemicolectomy depending on the location of the tumor. Most operations for colon cancer are accomplished by laparoscopic techniques with the exception of T4 tumors and tumors of the transverse colon, of which a laparotomy is used. If the lymph node status is positive, or there are any other signs of a high risk of recurrence, a patient is considered for adjuvant therapy by an oncologist.

Perioperative care includes the assessment and optimization of medical risk factors, thromboprophylaxis with low-molecular weight heparin and elastic compression stockings, standard anesthesia with epidural analgesia, avoidance of hypothermia and increased oxygen concentrations. Mechanical bowel preparation will be performed according to local hospital policies. In perioperative care the Enhanced Recovery after Surgery (ERAS) protocol will be followed.

### Data collection

All data will be collected prospectively using an electronic database designed for this study. The reasons for withdrawal, if any, will be documented. All data will be handled confidentially and pseudonymously.

For each participant, data collection will proceed as follows. The height and weight will be measured. BMI will be calculated. At baseline, the body weights three and six months previously will be obtained from the patients. The American Society of Anesthesiologists (ASA), WHO classification and Charlson Comorbidity Index will be recorded. History of smoking, comorbidities, additional supplement use and the patient’s medication will be recorded. The patient’s symptoms, date of first symptoms and date of diagnosis will be recorded. Whole-body computed tomography will be performed to assess the possibility of metastases or locally spread cancer and also to measure body composition. Colonoscopy findings will also be recorded.

The NRS-2002 score will be calculated on each time point. Each patient will score 2 points for disease severity at randomization and at the one-month follow-up visit because they have local cancer and upcoming or recent major abdominal operation. Bioimpedance analysis will be performed (Tanita 980) and the handgrip strength will be measured. Patients will keep a food diary (Supplementary file 1) for four days before the surgery and for four days at both one- and three- month time points after the operation to assess the energy and nutrient consumption. The actual consumption of the ONS will be recorded in both groups before and after the surgery.

Laboratory parameters including leukocytes, hemoglobin, C-reactive protein (CRP), carcinoembryonic antigen (CEA), creatinine and albumin concentrations will be measured. Cytokine levels will be examined. Samples of plasma, serum and whole blood will be saved at -80 °C for later analyses.

The surgical specimen will all be analyzed by a pathologist, and study pathologists will review the cancer type, grade and classification of malignant tumors (pTNMs) according to TNM8. In addition, the degree of inflammatory reaction at the tumor margin will be recorded using the Klintrup-Mäkinen score [36], and the number of lymphoid structures will be measured according to Väyrynen et al.’s method [37]. The number of intraepithelial lymphocytes and the SMI status will be recorded. The amount of tissue necrosis will be recorded [37]. Samples of subcutaneous and visceral fat tissue, rectus abdominis muscle, liver, tumor, healthy-looking bowel wall (including all layers), peritumoral bowel wall and stool samples will be collected during the operation. Samples will be taken for histological and biochemical analyses. In right-sided cancers, a sample from the terminal ileum will also be taken. The sample´s warm and cold ischemia times will be recorded.

A RAND-36 quality of life questionnaire and an exercise questionnaire (Supplementary file 2) will be completed at every time point [38].

Data about preoperative blood transfusions, oral iron use or intravenous iron infusions will be collected. Operation data will also be collected: operation code, duration, blood loss, infused units of red cells, infused amounts of crystalloids and colloids, bowel preparation before the operation, peroral and intravenous antibiotic use, vasopressor use during the operation, operating surgeon, anastomosis technique, stoma use, operation type and intraoperative complications.

Complications after the primary operation will be collected. Data concerning the length of stay in intensive care and total length of stay in hospital and discharge to home or institution will be collected. The date of first flatus, first passage of stool, use of ONS during hospital stay and ambulatory capacity during hospital discharge will be collected.

Complication data after the primary operation and after possible reoperations will be collected at every follow-up time point. The comprehensive complication index (CCI) will be analyzed. Registered complications include wound, respiratory, cardiovascular, gastrointestinal, anastomosis leakage, urogenital and renal, thromboembolic complications and other infections. The Clavien-Dindo classification will be determined for every complication.

The side effects of chemotherapy will be monitored, as well as discontinuation of chemotherapy. Information about cancer recurrence will be collected at every follow-up point.

### Follow-up

The follow-up protocol is presented in Table 2.

**Table 2:** Schedule of events

Schedule of events	1st meeting	Operation	Discharge	30 day	3 month phone call	1 year	2 year	5 year
Informed consent	x							
NRS-2002	x			x		x	x	x
Laboratory tests	x			x	x	x	x	x
Stool sample	x	x				x	x	x
CT scan	x					x		
BMI	x	x		x		x	x	x
Hand grip strength	x	x		x		x	x	x
Bioimpedance	x			x		x	x	x
Food diary	x			x	x			
QoL	x			x	x	x	x	x
Colonoscopy	x						x	x
Exercise questionnaire	x			x	x	x	x	x
Protocol deviation^a^	x^a^	x^a^	x^a^	x^a^	x^a^	x^a^	x^a^	x^a^
Complications and cancer recurrence		x^a^	x^a^	x^a^	x^a^	x^a^	x^a^	x^a^
Study exit form								x^b^

*Abbreviations*: *NRS-2002* Nutritional risk screening 2002, *CT* Computed Tomography, *BMI* Body Mass Index, *QoL* Quality Of Life

^a^ Complete if applicable

^b^ Complete when lost to follow-up, consent withdrawal, or when the subject has completed all study-related visits

#### First meeting and the operation

All patients attending this study will visit the outpatient clinic two to three weeks prior to the surgery. In the first meeting, patients are randomized by NRS-2002 as described. Bioimpedance and handgrip strength are measured. Laboratory tests, colonoscopy and CT scan results are registered. Patients will fill out a QoL questionnaire [38] and an exercise questionnaire (Supplementary file 2). Salivary samples are taken. A food diary (Supplementary file 1) is given to patients, and patients will return it when they present for their operation. The food diary will be checked upon return by a clinical nutritionist. All patients are given cancer patient nutrition guidelines. On arrival at the operation, BMI and handgrip strength will be measured. Data on patients’ ONS intake will be collected.

#### Postoperative care

Postoperative care follows the ERAS protocol. Operative and postoperative data are registered as described in data collection.

#### One-month follow-up visit and three-month phone call

The first follow-up visit is at the outpatient clinic 30 days after the surgery. At that time, the patient is interviewed about recovery from surgery. Patients whose prolonged recovery from surgery requires a stay in a primary care unit will be asked about the length of stay and possible complications after leaving the hospital. Data on possible readmissions and reoperations will be collected. Weights of the patients will be measured, the NRS-2002 score will be calculated, the bioimpedance analysis will be performed and the handgrip strength will be measured. The patients will complete the four-day food diary (Supplementary file 1), exercise questionnaire (Supplementary file 2) and QoL questionnaire [38]. Use of postoperative ONS is inquired from the patients. Blood samples are also taken.

The second follow-up at three months after the surgery, will be performed by phone. Albumin, CRP, CEA, hemoglobin and leucocyte count will be measured in health-care centers on a routine basis and the patients will complete a four-day food diary (Supplementary file 1) and QoL [38] and exercise questionnaire (Supplementary file 2). The patients will be interviewed about possible readmissions, reoperations and complications. The food diary will be checked by phone by a clinical nutritionist.

#### One-, two- and five-year follow-up visits

Patients will have follow-up visits one, two and five years after surgery. As is routine, CEA, hemoglobin and leucocyte count will be measured every three months for two years and then every six months until five years post-operatively. During the one-, two- and five-year follow-up visits, creatinine, albumin and CRP will also be measured. Samples of plasma, serum and whole blood will be stored at -80 °C for later analyses. Weight, BMI and bioimpedance analyses will be performed, the handgrip strength will be measured, and the NRS-2002 score will be calculated at each visit. The WHO classification, the Charlson comorbidity index and smoking habits will be recorded. The patients will also complete the QoL questionnaire [38] and exercise questionnaire (Supplementary file 2) at each time point. All patients will receive a whole-body CT scan one year after the surgery. CT scans monitoring the response to adjuvant chemotherapy will be utilized for analysis of recovery from sarcopenia and its effect on the survival and toxicity of chemotherapy. A colonoscopy will be performed two and five years after the surgery. During colonoscopy biopsy samples of normal looking ascending and descending colon and possible adenomas and tumors will be taken. A suction sample of effluent will be taken from the ascending colon. Additionally, a stool sample will be taken before bowel preparation. During every follow-up visit the patient will be interviewed about recovery, and the complications will be recorded. Data on cancer recurrence and its treatment will be collected.

### Study timeline

Randomization started on April 1, 2019 at the Oulu University Hospital and will last until 318 patients are recruited. Data will be collected until five years post-operatively.

### Statistical analysis

This is a superiority trial. Participants will remain in the groups to which they were originally assigned (intention to treat principal). Additionally, per protocol analyses will be performed as sensitivity analyses. In the per protocol analyses, protocol violation is deemed if a patient consumed < 50% of nutritional drinks in the intervention group or in control group if a patient drank more than 5 nutritional drinks.

All analyses will be adjusted by stratification variable (Nutritional status (NRS 2002) 2, 3–4 or 5). Additionally, the crude outcomes will be reported. The treatment effect will be assessed by odds ratios for categorical data, hazard ratios for survival data and mean differences for continuous data. Random effect models will be used in the case of repeatedly measured data (> 2 repeated measurements). If the incidence of outcome of interest is common (> 10%) then odds ratios will be converted to risk ratios [39].

The treatment effect will be calculated separately for each NRS 2002 stratification class (2, 3–4 and 5). However, as the sample size calculation was performed only for the total treatment effect, the results of the stratified analyses are hypothesis generating only.

## Ethical considerations

Patients participating in this study will be examined and treated with a standard protocol. Participation in this study is voluntary and will not affect the treatment. Patients were not involved in the design, or conduct, or reporting, or dissemination plans of the research. The results of this research will be published in compliance with the General Data Protection Regulations (GDPR).

There will not be any additional exposure to radiation due to this research. All imaging will be performed as a part of the routine treatment protocol. Tissue samples will be taken under general anesthesia during the cancer surgery and no additional pain will be induced. Harvesting of the samples may cause minor bleeding but not significant bleeding or surgical complications. Subcutaneous fat and muscle tissue will be taken from the operation field. All other samples will be taken from the resected bowel after the surgery.

There will be some additional laboratory examinations before and after the surgery. Additional blood sampling may cause minor pain but not significant complications. Bioimpedance analysis is a painless and noninvasive method to analyze body composition.

This research causes no extra costs to patients. The ONS is financed by research funding. There will be no support from the manufacturing industry.

This study follows the Declaration of Helsinki on medical protocols and ethics, and the study’s protocol has been approved by the Ethical Committee of Oulu University Hospital. Each participating hospital applied for study permission at their respective institutions. Important protocol modifications are communicated with the Oulu University Hospital Ethics Committee by amendments. All modifications are also registered at Clinical Trials.

Potential patients considered for participating in the research will give informed consent. Patients will be informed about this research in an outpatient clinic. They have the right to decline to participate without any impact on their treatment. Patients have the right to withdraw from the study without explanation at any time.

Patient confidentiality will be strictly maintained. Patients will be pseudonymised by study identification numbers, and all data will be handled without using names or personal social security numbers. Access to patient records will be limited to the study group and the investigator-delegated study coordinator.

## Discussion

This study uses a randomized trial to assess the efficiency of ONS two weeks before colon cancer surgery and 10 days after the operation compared to a control group that will not receive ONS. The hypothesis is that perioperative ONS will decrease the short-term and long-term postoperative morbidity and mortality rates of colon cancer patients undergoing curative surgery.

There are only a few studies of the effect of perioperative ONS on gastrointestinal cancer patients’ postoperative mortality and morbidity, and these studies have conflicting results. To our knowledge, there are no randomized controlled trials consisting of only colon cancer patients with a low to moderate risk of malnutrition having curative surgery. Previous studies have been performed on patients undergoing heterogenic types of gastrointestinal surgery with or without cancer [12–16, 18]. Nutritional support has also varied between studies with enteral and parental nutrition [18, 19]. Patients´ nutritional status was not taken into account during randomization [10, 12–14, 22]. In our study, patients will be randomized according to the NRS-2002 results. In this study, the patients´ nutritional status will also be measured with several other methods, including the hand grip strength, body composition and serum albumin, to determine which method would best forecast the treatment outcome. Previous studies have not analyzed the effect of perioperative nutritional support on long-term treatment outcomes.

European Society for Clinical Nutrition and Metabolism (ESPEN) guidelines for surgery recommend that nutritional status should be assessed before and after major surgery. It is recommended that patients with a risk of severe nutritional deficiencies receive seven to 14 days of nutritional therapy before an operation. ONS should be given to all malnourished cancer patients and high-risk patients before undergoing major abdominal surgery. Data on the impact of ONS are limited, and the results have been conflicting, especially concerning patients at low and moderate risks of malnutrition [5]. The ESPEN guidelines for cancer patients recommend that all cancer patients should be screened for the risk of malnutrition. These guidelines recommend that cancer patients who are malnourished or at risk of malnutrition should start nutritional intervention. ONS should be used if enriched diet is not sufficient. However, there is only moderate evidence behind this recommendation [40].

To our knowledge there are no randomized controlled trials that have studied the effect of a low-cost, easy-to-use perioperative oral supplement on postoperative mortality and morbidity in colon cancer patients with a low to moderate risk of malnutrition undergoing curative surgery. Published ESPEN guidelines on nutrition in cancer and surgery patients place a high emphasis on nutritional counseling and nutritional support when needed, but we are still missing evidence for these recommendations [40].

## Supplementary Information

**Additional file 1.** Food diary.

**Additional file 2.** Exercise questionnaire.

## Data Availability

Not applicable. The data sets generated and/or analyzed during the current study are not publicly available due to Finnish laws on privacy protection.

## Abbreviations

ONS: Oral nutritional support
NRS-2002: Nutritional risk screening 2002
QoL: Quality of life
eCRF: Case Report Forms
CT: Computed tomography
SMI: Skeletal muscle index
BMI: Body mass index
HU: Hounsfield unit
ERAS: Enhanced Recovery After Surgery
ASA: The American Society of Anesthesiologists
CRP: C-reactive protein
CEA: Carcinoembryonic antigen
TNM: Classification of malignant tumors
CCI: Comprehensive complication index
GDPR: The General Data Protection Regulations
ESPEN: European Society for Clinical Nutrition and Metabolism
