# Supplementary material for: Perioperative oral nutritional support for patients diagnosed with primary colon adenocarcinoma undergoing radical surgical procedures -Peri-Nutri Trial: study protocol for a randomized controlled trial
Source: BMC Nutr. 2022 Sep 2;8:89. doi: 10.1186/s40795-022-00591-y (PMC9438122; doi:10.1186/s40795-022-00591-y)
Supplement: Supplementary file 1 — Additional file 1. Food diary. [file 40795_2022_591_MOESM1_ESM.docx]

# Instructions for keeping the food diary

In the food diary, record all the food and drink that you have consumed over four days. Write in the diary immediately after you eat, including small snacks. Include at least one holiday in the diary (e.g., on the weekend or another day if your food consumption differs on holidays, weekends, and weekdays).

Estimate the amounts by piece, deciliter (dl), tablespoon, teaspoon, or slice. Include the method of cooking and the kind of fat used.

**FOOD DIARY Day 1**

Name______________________________________

Date________________________________

In this table, write the time when you ate and what you ate. Estimate the amounts of food and drink you consumed.

| Time | Food and Drink | Amount (pieces, dl, g, glasses, etc.) |
| --- | --- | --- |
|  |  |  |
|  |  |  |
|  |  |  |
|  |  |  |
|  |  |  |
|  |  |  |
|  |  |  |
|  |  |  |
|  |  |  |
|  |  |  |
|  |  |  |
|  |  |  |
|  |  |  |
|  |  |  |
|  |  |  |
|  |  |  |
|  |  |  |
|  |  |  |

**FOOD DIARY Day 2**

Name______________________________________

Date________________________________

In this table, write the time when you ate and what you ate. Estimate the amounts of food and drink you consumed.

| Time | Food and Drink | Amount (pieces, dl, g, glass etc.) |
| --- | --- | --- |
|  |  |  |
|  |  |  |
|  |  |  |
|  |  |  |
|  |  |  |
|  |  |  |
|  |  |  |
|  |  |  |
|  |  |  |
|  |  |  |
|  |  |  |
|  |  |  |
|  |  |  |
|  |  |  |
|  |  |  |
|  |  |  |
|  |  |  |
|  |  |  |

**FOOD DIARY Day 3**

Name______________________________________

Date________________________________

In this table, write the time when you ate and what you ate. Estimate the amounts of food and drink you consumed.

| Time | Food and Drink | Amount (pieces, dl, g, glasses, etc.) |
| --- | --- | --- |
|  |  |  |
|  |  |  |
|  |  |  |
|  |  |  |
|  |  |  |
|  |  |  |
|  |  |  |
|  |  |  |
|  |  |  |
|  |  |  |
|  |  |  |
|  |  |  |
|  |  |  |
|  |  |  |
|  |  |  |
|  |  |  |
|  |  |  |
|  |  |  |

**FOOD DIARY Day 4**

Name______________________________________

Date________________________________

In this table, write the time when you ate and what you ate. Estimate the amounts of food and drink you consumed.

| Time | Food and Drink | Amount (pieces, dl, g, glasses, etc.) |
| --- | --- | --- |
|  |  |  |
|  |  |  |
|  |  |  |
|  |  |  |
|  |  |  |
|  |  |  |
|  |  |  |
|  |  |  |
|  |  |  |
|  |  |  |
|  |  |  |
|  |  |  |
|  |  |  |
|  |  |  |
|  |  |  |
|  |  |  |
|  |  |  |
|  |  |  |
