# Supplementary material for: Perioperative oral nutritional support for patients diagnosed with primary colon adenocarcinoma undergoing radical surgical procedures -Peri-Nutri Trial: study protocol for a randomized controlled trial
Source: BMC Nutr. 2022 Sep 2;8:89. doi: 10.1186/s40795-022-00591-y (PMC9438122; doi:10.1186/s40795-022-00591-y)
Supplement: Supplementary file 2 — Additional file 2. Exercise questionnaire. [file 40795_2022_591_MOESM2_ESM.docx]

EXERCISE QUESTIONNAIRE

1. What is the physical workload in your current work? Choose one of alternatives 1–4 (If you are not at work, select number one).

1. Light sedentary work, only light manual tasks (watchmaker, office job, industry sewer, etc.)
2. Moving work without heavy work movements or carrying heavy loads (supervisor, shop assistant, moving office work, etc.)
3. Moving work with a lot of carrying or climbing a lot of stairs.

(e.g., carpenter, heavy industrial worker, etc.)

1. Heavy manual work, constant lifting or carrying heavy loads, digging, shoveling, or hammering. (e.g., lumberman, heavy agricultural worker, construction worker, etc.)

2. How often do you participate in physical activity/ exercise during your leisure time? If it depends on the season, select an option to describe your average situation.

1. During my leisure time, I read, watch television, and do light housework.
2. During my leisure time, I walk, bicycle, or do other activities at least four hours a week. Include walking, fishing, hunting, easy gardening, etc. but not commuting).
3. During my leisure time, I practice physical exercises, such as running, jogging, skiing, gym, swimming, ball games, heavy gardening, or other equivalent activity at least three hours a week.
4. During my leisure time, I regularly practice competitive sports, such as running, orienteering, skiing, swimming, ball games, or other physically demanding sports several times a week.

3. If you answered 3 or 4 at last question, mark which type of physical activity you prefer to practice and how many hours you practice a week? __________________________________________

______________________________________________________________________________

______________________________________________________________________________

______________________________________________________________________________

4. How many minutes do you commute to work by walking, bicycling, or in other ways that require physical exercise? (Include the amount of time it takes you to go to work and return home.)

1. I do not work or commute to work using a motor vehicle.
2. Less than 15 minutes a day
3. 15–29 minutes a day
4. 30–44 minutes a day
5. 45–59 minutes a day
6. More than one hour a day

5. How often do you practice leisure-time sports for at least 20 minutes so that you at least get slightly out of breath or sweat? Do not include commuting to work.

1. Less than once a week
2. Once a week
3. Two times a week
4. Three times a week
5. Four times a week
6. Five times a week
7. Six times a week or more

6. How long do you usually practice leisure-time physical exercise at a time?

1. 15–29 minutes
2. 30–59 minutes
3. 60–89 minutes
4. 90 minutes or longer.

7. How many minutes a day do you walk, bicycle, or do some other physical exercise during your leisure time (e.g., gardening, house cleaning, repairing, shopping)? No dot include commuting to work or leisure-time physical exercise.

1. Less than 15 minutes a day
2. 15–29 minutes a day
3. 30–44 minutes a day
4. 45–59 minutes a day
5. More than one hour a day

8. How would you describe your level of physical fitness?

1. Excellent
2. Good
3. Fair
4. Poor
5. Very poor

9. Approximately how many hours during a weekday do you spend sitting ? Mark 0 if none.

1. During work at the office or equivalent _____ h_____ min
2. At home watching TV or videos _____ h_____ min
3. At home at the computer _____ h_____ min
4. In a vehicle _____ h_____ min
5. Elsewhere _____ h_____ min
